# Lipid environment determines the drug-stimulated ATPase activity of P-glycoprotein

**DOI:** 10.3389/fmolb.2023.1141081

**Published:** 2023-02-23

**Authors:** Nghi N. B. Tran, A. T. A. Bui, Valeria Jaramillo-Martinez, Joachim Weber, Qinghai Zhang, Ina L. Urbatsch

**Affiliations:** ^1^ Department of Cell Biology and Biochemistry, Texas Tech University Health Sciences Center, Lubbock, TX, United States; ^2^ Center for Membrane Protein Research, Texas Tech University Health Sciences Center, Lubbock, TX, United States; ^3^ Department of Chemistry and Biochemistry, Texas Tech University, Lubbock, TX, United States; ^4^ Department of Integrative Structural and Computational Biology, The Scripps Research Institute, La Jolla, CA, United States

**Keywords:** P-glycoprotein, multidrug ABC transporter, verapamil-stimulated ATPase activity, membrane lipid composition, artificial plasma membrane mimic, mixed detergent/lipid micelles, liposomes, nanodiscs

## Abstract

P-glycoprotein (Pgp) is a multidrug transporter that uses the energy from ATP binding and hydrolysis to export from cells a wide variety of hydrophobic compounds including anticancer drugs, and mediates the bioavailability and pharmacokinetics of many drugs. Lipids and cholesterol have been shown to modulate the substrate-stimulated ATPase activity of purified Pgp in detergent solution and the substrate transport activity after reconstitution into proteoliposomes. While lipid extracts from *E. coli*, liver or brain tissues generally support well Pgp’s functionality, their ill-defined composition and high UV absorbance make them less suitable for optical biophysical assays. On the other hand, studies with defined synthetic lipids, usually the bilayer-forming phosphatidylcholine with or without cholesterol, are often plagued by low ATPase activity and low binding affinity of Pgp for drugs. Drawing from the lipid composition of mammalian plasma membranes, we here investigate how different head groups modulate the verapamil-stimulated ATPase activity of purified Pgp in detergent-lipid micelles and compare them with components of *E. coli* lipids. Our general approach was to assay modulation of verapamil-stimulation of ATPase activity by artificial lipid mixtures starting with the bilayer-forming palmitoyloyl-phosphatidylcholine (POPC) and -phosphatidylethanolamine (POPE). We show that POPC/POPE supplemented with sphingomyelin (SM), cardiolipin, or phosphatidic acid enhanced the verapamil-stimulated activity (V_max_) and decreased the concentration required for half-maximal activity (EC_50_). Cholesterol (Chol) and more so its soluble hemisuccinate derivative cholesteryl hemisuccinate substantially decreased EC_50_, perhaps by supporting the functional integrity of the drug binding sites. High concentrations of CHS (>15%) resulted in a significantly increased basal activity which could be due to binding of CHS to the drug binding site as transport substrate or as activator, maybe acting cooperatively with verapamil. Lastly, Pgp reconstituted into liposomes or nanodiscs displayed higher basal activity and sustained high levels of verapamil stimulated activity. The findings establish a stable source of artificial lipid mixtures containing either SM and cholesterol or CHS that restore Pgp functionality with activities and affinities similar to those in the natural plasma membrane environment and will pave the way for future functional and biophysical studies.

## Introduction

P-glycoprotein, also known as MDR1 or ABCB1, is a plasma membrane protein that can pump a wide range of hydrophobic, structurally diverse substances out of cells (Chufan et al., 2015). The Pgp substrates include many chemotherapeutic drugs used for the treatment of cancer, HIV/AIDS, neurodegenerative and cardiovascular diseases (Al-Khazaali et al., 2014; Waghray and Zhang, 2018; Jia et al., 2020). Pgp participates in the transepithelial transport of drugs and metabolites which makes it an important determinant in the absorption, distribution, metabolism and excretion (ADME) that affect the pharmacokinetics and drug-drug interactions of therapeutics across many diseases (Giacomini et al., 2010; Yee et al., 2018). Pgp is also richly expressed at several blood-organ barriers including the brain and placenta where it protects those sanctuaries from harmful xenobiotics, environmental pollutants, and waste products of cellular metabolism (Storelli et al., 2021). Additionally, Pgp is a key player in multidrug resistance (MDR) of cancers (Robey et al., 2018). Therefore, it is critical to comprehend the mechanism by which Pgp recognizes and extrudes medicines in the context of the tissue microenvironment.

Pgp is an ATP-binding cassette (ABC) transporter that consists of two nucleotide binding domains (NBDs) that energize transport by binding and hydrolyzing ATP, and two transmembrane domains (TMDs) that harbor the drug binding site(s) within the lipid bilayer. A large hydrophobic binding surface in the center of the transmembrane domains was shown in Pgp structures determined by X-ray crystallography and cryo-electron microscopy (cryo-EM). Pgp structures have been solved with multiple bound substrates, such as QZ-59 cyclopeptides, vincristine, taxol, and as well as with inhibitors elacridar, tariquidar, zosuquidar (Aller et al., 2009; Szewczyk et al., 2015; Alam et al., 2018; Alam et al., 2019; Nosol et al., 2020). Because Pgp substrates are generally hydrophobic, they are likely extruded from within the membrane rather than from the aqueous phase. Indeed, Pgp is thought to act as “hydrophobic vacuum cleaner” (Gottesman and Ling, 2006) that intercepts hydrophobic compounds from the inner leaflet of the membrane bilayer before they reach the cytoplasm and pumps them back out of cells. As a membrane transporter and because of the intricate interactions of the hydrophobic substrate with the bilayer, the surrounding lipid environment is an important regulator of Pgp activity (Sharom, 2014; Stieger et al., 2021).

### Phospholipids modulate Pgp function

Early studies of Pgp in native plasma membranes fractions from mammalian cells showed robust stimulation of the ATPase activity by transport substrates like verapamil and vinblastine (al-Shawi and Senior, 1993). Purification of Pgp in detergents often led to reduced ATPase activity but could be restored with the supplementation of lipids (e.g., tissue extracts from *E. coli*, sheep or bovine brain, bovine liver, for available lipid composition see Table 1). However, the basal activity (in the absence of added drug) and the drug-stimulated activities varied considerably depending on the detergent used for solubilization and the lipid source (Ambudkar et al., 1992; Sharom et al., 1993; Urbatsch and Senior, 1995). Similar studies with extracts of defined phospholipids or synthetic lipids including phosphatidylcholine (PC), -ethanolamine (PE), -serine (PS) and -inositol (PI); showed that they all supported basal ATPase activity of purified Pgp and protected its thermal inactivation (Doige et al., 1993; Yang et al., 2017). This may be due to specific phospholipid binding to the outer surface of Pgp, which may affect the protein stability and activity (Marcoux et al., 2013).

**TABLE 1:** Lipid composition of native mammalian membranes.

	*E. coli* polar lipid extract	Bovine liver polar lipid extract	Porcine brain polar lipid extract	Mammalian total cell lipid^a^ ^)^	Human liver microsomes	Rat liver plasma membrane^b^	Plasma membrane mimic
Phosphatidylcholine (PC)		42	13	45 to 55 ^b^	42	36 to 41	∼40
Phosphatidylethanolamine (PE)	67	26	22	15 to 25	31	23 to 26	∼24
Phosphatidylserine (PS)		10	19	2 to 10	5	9 to 10	∼9
Phosphatidylinositol (PI)			4	10 to 15	13	4 to 8	^c^
Phosphatidylglycerol (PG)	23						
Sphingomyelin (SM)				5 to 10	4	16 to 25	∼22
Cardiolipin	10			2 to 5			
PA, BMP			1	1 to 2		0.5	
Residual		17 ^d^	31 ^d^			1 to 2	
Cholesterol		5		10 to 20	7		
Cholesterol:phospholipid						ratio 1:1^e^	ratio 1:1
References	Avanti Polar Lipids	Avanti Polar Lipids	Avanti Polar Lipids	Vance and Steenbergen (2005)	Kapitulnik et al. (1987)	Zambrano et al. (1975)	This study
						van Meer et al. (2008)	

^a^ Cultured cell extracts from a typical mammalian cell (Vance and Steenbergen, 2005); examples from HEK, CHO, MDCK, and other cell lines can be found in (Symons et al., 2021).

^b^ Original references are from rat plasma membranes; these are considered similar to human plasma membranes (Ingólfsson et al., 2014; Domicevica et al., 2018; Symons et al., 2021).

^d^ Residuals are not specified on the Avanti Polar Lipids website.

^e^ Values are given in % (w/w); cholesterol is often reported as a weight ration relative to total phospholipid content. A ratio of 1:1 amounts to 50% total cholesterol (w/w).

^c^ Was not include due to inhibitory effect, see text.

It has been suggested in the literature that phospholipids themselves are substrates for Pgp (Rothnie et al., 2001). Sharom et al. detected the flippase activity of Pgp using the NBD fluorescence-labeled PC, PE and PS derivatives, reviewed in (Sharom, 2014). Albeit this flippase activity is much lower than the dedicated PC-lipid flippase activity of its close relative MDR2 (ABCB4) both of which are prominent in the liver canalicular membrane duct, where they work together with cholesterol transporter ABCG5/G8 and the bile salt export pump ABCB11 to excrete hydrophobic drugs, lipids and cholesterol dissolved in bile acids (Neumann et al., 2017).

### Cholesterol modulates bilayer properties and Pgp function

Cholesterol is one of the key components in animal cell membranes. It influences membrane properties such as membrane packing, elasticity and fluidity, affects lateral and transmembrane diffusion and overall stabilizes the structure of phospholipid bilayers. Over the years, a connection between Pgp activity and cholesterol has evolved. Pgp ATPase activity has been reported to be affected by the presence of lipid and cholesterol (Sharom, 2008). Besides its effect on Pgp transport activity, cholesterol may alter the availability of hydrophobic drugs in the membrane (Rothnie et al., 2001; Modok et al., 2004; Belli et al., 2009). Interestingly, ordered POPE and cholesterol molecules have been found in cryo-EM structures tightly associated with human Pgp in nanodiscs composed of brain lipid extract supplemented with cholesterol (Alam et al., 2019). On the contrary, phospholipids and cholesterol have not been revealed in the internal cavity of existing high-resolution Pgp structures. Hence the evidence for Pgp as a lipid or cholesterol transporter is still lacking. More likely, the membrane lipids modulate the conformational changes associated with drug binding and transport.

We previously have reconstituted Pgp into *E. coli* lipid nanodiscs that displayed high verapamil-stimulated ATPase activity. These nanometer-scale discoidal structures contain a phospholipid bilayer encased by membrane scaffold proteins (MSPs), which are soluble and stable in aqueous solutions (Zoghbi et al., 2017). Although nanodiscs differ from native membranes in physical properties such as curvature, and in the complexity of their lipid composition, they are excellent lipid–bilayer platforms for many biophysical applications including optical measurements that require reduced light scattering (Ritchie et al., 2009). With access from both sides, nanodisc allows the protein to interact with the substrate from different directions. However, tissue extracts of lipids, including *E. coli* lipids, carry very high fluorescence in the tryptophan excitation/emission (290/345 nm) range and are not suitable for biophysical studies using the intrinsic fluorescence of Pgp’s eleven native tryptophans.

#### Phospholipid and cholesterol composition of membranes

The composition of lipid components in different organelles and tissues varies greatly; thus, it is traditionally difficult to assess the endogenous activity of Pgp. In a typical mammalian cell, the total membrane lipid composition consist of roughly 45%–55% PC, 15%–25% PE, 5%–10% PS, 10%–15% PI, 5%–10% sphingomyelin (SM), and 10%–20% cholesterol (Table 1) (Vance and Steenbergen, 2005; van Meer et al., 2008). Human liver microsomes, which contain a mixture of endoplasmic reticulum, Golgi and plasma membranes, generally have a higher content of about 31% PE and lower relative contents of SM and cholesterol. In contrast, plasma membranes are enriched in total SM (>15%) and cholesterol (ratio is approximately 1:1 cholesterol:phospholipid, w/w) accounting for its greater rigidness (Symons et al., 2021). Furthermore, within the plasma membrane SM (up to 26%) and cholesterol are accumulating in detergent-insoluble microcompartments known as lipid rafts, in which Pgp tends to gather (Ismair et al., 2009; Hullin-Matsuda et al., 2014). In both microsomes and plasma membranes, PC is the most abundant phospholipid (36%–42%), followed by PE and SM with lower PS and PI contents (Table 1).

In this study, we measure the effects of individual lipids and lipid mixtures on the basal and verapamil-stimulated ATPase function of Pgp. We use both natural lipids and defined synthetic lipids to identify those that promote Pgp activity. We further probe increasing concentrations of supplemental cholesterol and its more soluble hemisuccinate derivative CHS and demonstrate favorable interaction of CHS on the Pgp ATPase function. By these measurements, we establish a stable source of artificial lipid mixtures containing POPC, POPE, and cholesterol/CHS that can support Pgp ATPase activity, and optimize the reconstitution of Pgp into lipid nanodiscs with high yields and high activity. Our study reassures that the phospholipid and cholesterol composition play a crucial role in the basal and drug-stimulated ATPase activity affecting both the apparent binding affinity for verapamil and the V_max_.

## Methods and materials

### Materials

Protein kinase A, lactate dehydrogenase, and phosphoenol-pyruvate were purchased from Roche CustomBiotech (Indianapolis, IN). Adenosine-5′-triphosphate disodium salt (ATP) ultrapure 98% was obtained from Alfa Aesar (Tewksbury, MA). Verapamil was acquired from Sigma Aldrich (Saint Louis, MO). n-dodecyl-β-D-maltopyranoside (DDM) was bought from Inalco S. p.A (Milano, Italy). Nicotinamide adenine dinucleotide (NADH) was purchased from Sigma-Aldrich (Burlington, MA).

*E. coli* polar lipids (polar extract) and synthetic lipids were acquired from Avanti (Alabaster, AL); these include 1-palmitoyl-2-oleoyl-sn-glycero-3-phosphocholine or 16:0-18:1 PC (POPC), 1-Palmitoyl-2-oleoyl-sn-glycero-3-phosphatidylethanolamine (POPE), 1-palmitoyl-2-oleoyl-sn-glycero-3-phospho-L-serine (POPS), 1-palmitoyl-2-oleoyl-sn-glycero-3-phosphatidylinositol (POPI), 1-Palmitoyl-2-oleoyl-sn-glycero-3-phosphatidylglycerol (POPG), DPPA, 1,2-dipalmitoyl-sn-glycero-3-phosphate or 16:0 PA, 1,2-dimyristoyl-sn-glycero-3-phosphocholine (DMPC). Sphingomyelin (SM) was >99% pure from porcine brain with major acyl chains of 18:0 (50%) and 21:1 (21%), and cardiolipin (CL) was from >99% bovine heart with major acyl chains of 18:2 (90%). All synthetic lipids, SM and CL had very low tryptophan fluorescence (ex/em 295/350 nm) if purchased as powder. Cholesterol (Chol) and cholesteryl hemisuccinate (CHS) were purchased from Anatrace (Maumee, OH).

General chemicals were at the highest grade from Thermo Fisher Scientific (Waltham, Massachusetts).

### Tryptophan-free membrane scaffold protein mutagenesis

The membrane scaffold protein MSP1E3D1 plasmid was a gift from Stephen Sligar (Addgene plasmid # 20066; http://n2t.net/addgene:20066; RRID:Addgene_20066) obtained from Addgene (Denisov et al., 2009). The three native tryptophans (Trps) were substituted by PCR mutagenesis (W41R/W77Q/W143Q) to create a Trp-less MSP1E3D1 named WL-MSP. Wild-type and WL-MSP were expressed in BL21Gold (DE3) *E. coli* cells in Terrific broth with 70 μg/ml kanamycin after induction with 1 mM IPTG for 2–4 h at 37°C as described (Ritchie et al., 2009). The N-terminal hepta histidine-tag was used for purification by established protocols (Ritchie et al., 2009) with the following modifications: Because Triton-X100 has a very high absorption around 280 nm, 1% DDM was substituted for Triton during cell breakage. WL-MSP bound to Ni-NTA resin was first washed with buffer M (40 mM TrisCl pH = 8, 300 mM NaCl) containing 50 mM sodium cholate (10 column volumes (cv)), followed by 50 mM sodium cholate buffer M containing 20 mM imidazole (10 cv). Then cholate was washed off with buffer M containing 0.1% DDM (10 cv), followed by a stringent wash in detergent-free buffer (10 cv) containing 50 mM imidazole to remove tightly bound contaminants. The WL-MSP protein was eluted in detergent-free buffer M with 400 mM imidazole, dialyzed against buffer M and frozen in aliquots. Protein concentration was determined by its absorption at 280 nm using a molar extinction coefficient Ɛ of 11,520 M^-1^cm^-1^ with 1 absorption unit containing 2.84 mg/ml protein.

### Lipid mixtures of varied composition

Lipids from Avanti Polar Lipids were obtained as lyophilized powder. Calculated amounts of lipid powder were weighed into a glass vial and pumped under vacuum at ≤20 microns for 16–20 h at room temperature to remove trace amounts of solvent. Lipid mixtures were suspended into 50 mM TrisCl buffer pH, 7.4 at a final concentration of 20 mg/ml by rotating for at least 8–10 h at room temperature, followed by several freeze cycles at −80°C followed by thawing at room temperature. Lipid powder and suspension were kept under inert nitrogen gas at all times and stored frozen at −80°C. For nanodisc reconstitutions, lipid mixtures were dissolved in DDM at a ratio of 4:1 DDM to lipid (w/w) unless otherwise indicated. For lipid mixtures containing CHS, the indicated amount of phospholipids and CHS powder were weighted prior to dissolving in TrisCl buffer.

### Protein purification and quantification

Mouse mdr1a Pgp (codon-optimized abcb1a, GenBank JF834158) was expressed in *Pichia pastoris*, and grown in fermentor cultures as previously described (Bai et al., 2011). Tobacco, Etch Virus protease (TEV)-cleavable Twin-Strep and His_6_ –tags were engineered to the C-terminus to facilitate purification by tandem affinity chromatography on Ni-NTA and Strep-Tactin resins as described (Swartz et al., 2020), with the following modifications. Microsomal membrane preparations were solubilized at a concentration of 2–3 mg/ml keeping a constant ratio of detergent to protein of 4:1 (w/w). All purification buffers contained 50 mM Tris pH 8, 10% glycerol, and 500 mM NaCl (Buffer A). Detergent concentrations were reduced to 0.05% DDM during chromatography. Pgp was eluted from the Strep-Tactin superflow resin (Qiagen, Valencia, CA) in the presence of 1 mM DTT, 1 mM TCEP by competition with 4 mM desthiobiotin.

The concentrations of purified Pgp preparations were initially determined from the absorbance at A280 nm using a calculated molar extinction coefficient Ɛ including the purification tags of 126,630 M^-1^cm^-1^. Protein concentrations were verified by the bicinchoninic acid (BCA) protein assay using BSA as a standard. Finally, increasing protein amounts of a Pgp standard and newly purified Pgps were resolved side-by-side on SDS-PAGE gels, stained with Coomassie Brilliant Blue, and the Pgp bands quantified using ImageJ (http://rsbweb.nih.gov) to compare mutant Pgp levels between purifications, and after reconstitutions. From 100 g of cells, we routinely purified 6–7 mg of highly pure Pgp at the concentration of 0.8–1 mg/mL. Pgp was frozen in aliquots of 100–200 μl at −80°C.

### ATPase activity measurements

To determine the ATPase activity, purified Pgp (∼100 µL) in detergent was activated by incubation with 10 mM DTT and an equal volume of 20 mg/ml of lipids added, giving a final concentration of 1% (w/v) lipids for 15 min at 4°C. Then the protein concentration was adjusted with TrisCl buffer to 0.075 μg/μl 0.5–1.0 µg (∼10 µl) samples were added to 200 µl of ATP cocktail in 96-well plates. The rate of ATP hydrolysis was measured at 37°C in an enzyme linked continuous optical assay utilizing an ATP regeneration system (Urbatsch et al., 1995; Urbatsch et al., 2000), in the absence and presence of increasing concentrations of verapamil. Purified Pgp mixed with *E. coli* polar lipids typically displayed a specific ATPase activity at 30 µM Verapamil of 3.8–4.0 μmol/min/mg.

Verapamil stocks (50 mM) and 2x-serial dilutions were made in water, 2 µl of the serial dilutions were added to 200 µl of ATP cocktail. ATP activity was monitored for 20 min to 2 h during which the slopes were constant. Statistical analyses were done as described (Swartz et al., 2013; Swartz et al., 2014). EC_50_ values were calculated from fits according to f = V_b_+((V_max_-V_b_)*X^b^/(Ks^b^ + X^b^)), where Vb is the basal activity (in the absence of verapamil), V_max_ is the maximum activity, X is the concentration of verapamil, b is the Hill coefficient of the upward curve, Ks is the concentration for half-maximal stimulation or EC_50_. For each data fit, *R* ^2^ was greater than 0.97 and each of the parameters was statistically significant (*p* < 0.05). Lines in the graphs represent fits to the data points (open and closed symbols) using the following equation f = V_b_+((V_max_-V_b_)*X^b^/(K_s_^b^ + X^b^))+(V_max_-(V_max_-V∞)*X^m^/(K_i_^m^ + X^m^)), where Vb is the basal activity (in the absence of verapamil), V_max_ is the maximum activity, X is the concentration of verapamil, b is the Hill coefficient of the upward curve, Ks is the concentration for half-maximal stimulation or EC_50_, V∞ is the activity at infinite verapamil concentrations, m is the Hill coefficient of the downward curve, and Ki is the concentration for half-maximal inhibition or IC_50_. All statistical analyses were performed with Sigmaplot 11.

### Protein reconstitution and quantification

For liposome reconstitutions, purified Pgp in detergent was activated with 10 mM DTT and lipid mixture at a ratio of 1:250 Pgp:lipids (mol/mol). The sample was then diluted with 50 mM TrisCl buffer to give a final glycerol concentration of 4%.

For nanodisc reconstitution, purified Pgp in detergent was activated with 10 mM DTT before adding membrane scaffold protein (MSP) and lipid mixture at a ratio of 1:5:250 Pgp:MSP:lipids (mol/mol/mol). The sample then diluted using 50 mM TrisCl, buffer to give a final glycerol concentration of 4%. For both liposome and nanodisc reconstitutions, for every 1 mg of purified protein, 2 mg of Bio-Beads were added in two additions to remove the detergent. First addition consisted of 1/3 of the total Bio-Beads then placed on a rocker for 2 h at 4°C before adding the second batch of Bio-Beads with continuous rocking for 16–18 h at 4°C. Bio-Beads were removed by filtration through a chromatography column (BioRad) and washed with twice the initial volume of 50 mM TrisCl, 150 mM NaCl, 2% glycerol (buffer M). The filtrate was centrifuged for 15 min at 12,000 g and 4°C to pellet precipitated protein. The supernatant contained a mixture of Pgp-discs and “empty” lipid-nanodiscs that had not entrapped Pgp. This mixture can be directly used for ATPase activity measurement of Pgp since empty nanodiscs show zero activity. Pgp liposomes were directly used for ATPase assays and were not further purified.

Freshly prepared proteoliposomes were snap-frozen at 80°C in small aliquots and assayed within 2 days. Aliquots were thawed at room temperature, and then 2.5–5 µl were diluted into 200 ul of ATPase cocktail, and the ATP hydrolysis monitored at 37°C as above. To test the orientation of the NBDs in the proteoliposomes, aliquots were incubated overnight at 4°C on a rotator without or with TEV protease that cleaves the C-terminal Strep-tag of only those Pgp molecules with the NBDs accessible facing outside of sealed proteoliposomes. Loss of the Strep-tag was detected on Western blots with the anti-Strep tag antibody (Qiagen) and the SuperSignal™ West Pico enhanced chemiluminescent (ECL) substrate; luminescence signals were quantitated with the ImageQuant software.

For separation of Pgp-discs and empty nanodiscs, we took advantage of the Twin-Strep tag on Pgp to bind the protein to Strep-Tactin resin and wash off empty nanodiscs in detergent-free buffers. For this, 1 mg Pgp reconstituted as detailed above was incubated with 1 ml of Strep-tactin resin in 1.5 ml total volume by rotating in a sealed chromatography column for 5 h at 4°C. The flow-through was collected in the same column, and unbound empty nanodiscs were washed off with 10 column volume of size exclusion chromatography (SEC) buffer (20 mM HEPES pH 7.4, 150 mM NaCl, 4% glycerol). The Pgp-discs were eluted off the resin by cleavage of the tag. For this TEV protease was added to the resin at a Pgp:TEV ratio of 4:1 (w/w) in a total of one cv by rotating in the same column for 16–18 h at 4°C to allow for optimal cleavage. The flow through was collected and the Strep-tactin resin was washed with 2 cv of SEC buffer to collect all cleaved sample and combined with the column elution.

Isolated Pgp-discs were analyzed for purity and Pgp concentration on Coomassie-stained SDS-gels and cross-referenced with ATPase activity. The Pgp protein bands were quantified by comparison with a purified Pgp standard using ImageJ. We routinely obtained Pgp concentration of 0.04–0.05 μg/μl; total protein recovery was variable between experiments and was about 25% ± 6% of the starting Pgp using POPC/POPE/Chol/CHS lipid mixtures. For ATPase assays, about 20 µL Pgp-disc samples were mixed with 200 µl ATP cocktail containing 30 µM verapamil and incubated for 50 min to 2 h at 37°C, and ATPase activity calculated as above. ATPase measurements of samples “Before” and “After” separation on Strep-Tactin resin were assayed in triplicates, and the total ATPase activity calculated per input Pgp.

In all cases, data are expressed as means of at least two independent reconstitutions ± range. Graphs were plotted using SigmaPlot 11.0 from Systat Software, Inc., San Jose California United States, www.systatsoftware.com. Statistical analyses were performed using GraphPad Prism 9 Software (San Diego, California United States). Statistical differences between groups were analyzed by two-tailed unpaired Student’s t-test, or by one-way analysis of variance (ANOVA) followed by Dunnett’s, Bonferroni’s, or Tukey’s test, for single and multiple comparisons; a *p* < 0.05 was considered significant. For these statistical tests, the normality was confirmed using the GraphPad Prism 9 software.

## Results

### Parameters analyzed to evaluate Pgp function

The overarching goal of this study was to compare Pgp functionality in different lipid environments. For this purpose, we measured V_max_ and, in order to assess the affinity for transport substrates, EC_50_ for verapamil-induced ATPase activity. In some cases with a high basal activity (i.e., in absence of verapamil), the additional activation by verapamil was too small to reliably determine EC_50_. In those cases, it was easier to determine the verapamil concentration necessary for maximal ATPase activity.

### Pgp ATPase activity in *E. coli* lipid

**FIGURE 1:**
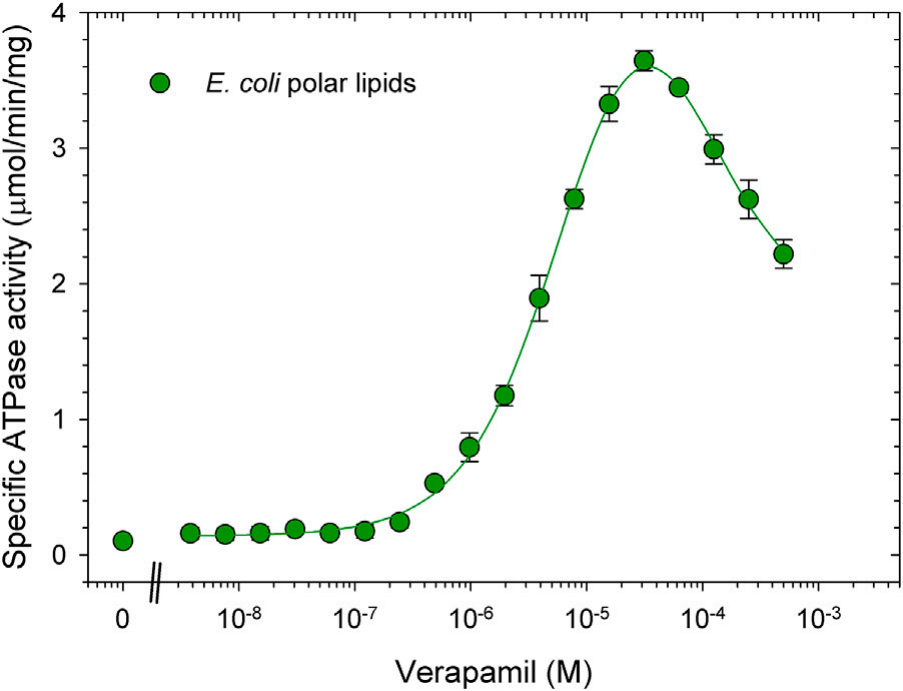
Verapamil-induced stimulation of ATPase activity in DDM-*E. coli* lipid mixed micelles. Purified Pgp in detergent solution was activated with 1% *E. coli* polar lipids and 10 mM DTT as described in experimental procedures. ATPase activity was measured in the presence of increasing concentrations of verapamil as indicated. Data points represent the mean ± SEM (*n* = 3). Kinetic parameters obtained from a fit (solid line) to the data points is listed in Table 2, for details see Methods.

The ATPase activity of Pgp was previously measured using *E. coli* polar lipid extract (Avanti Polar Lipids) (Bai et al., 2011; Shukla et al., 2017). Typically with *E. coli* lipids, purified Pgp in DDM was activated by simply mixing the protein in detergent solution (usually 0.1% DDM) with lipids, together with 10 mM DTT to fully reduce inhibitory disulfide bonds (Urbatsch et al., 2001). The highest activation of ATPase by verapamil in this detergent/lipid mixture was reported at around 30 μM, with a V_max_ of 3.8 μmol/min/mg and an EC_50_ of around 3 µM. (Figure 1 **,** see also (Urbatsch et al., 2000; Bai et al., 2011). Basal ATPase activity in the absence of verapamil was low at around 0.15 μmol/min/mg. In dose-response curves for verapamil activation of ATPase activity we have consistently observed a Hill coefficient of greater than 1.2 suggesting two binding sites that interact with positive cooperativity. Moreover, a negative slope of the curve at high concentrations of verapamil suggests (an) secondary inhibitory site(s) with a lower affinity for verapamil.

### Pgp ATPase activity in defined phospholipids

**FIGURE 2:**
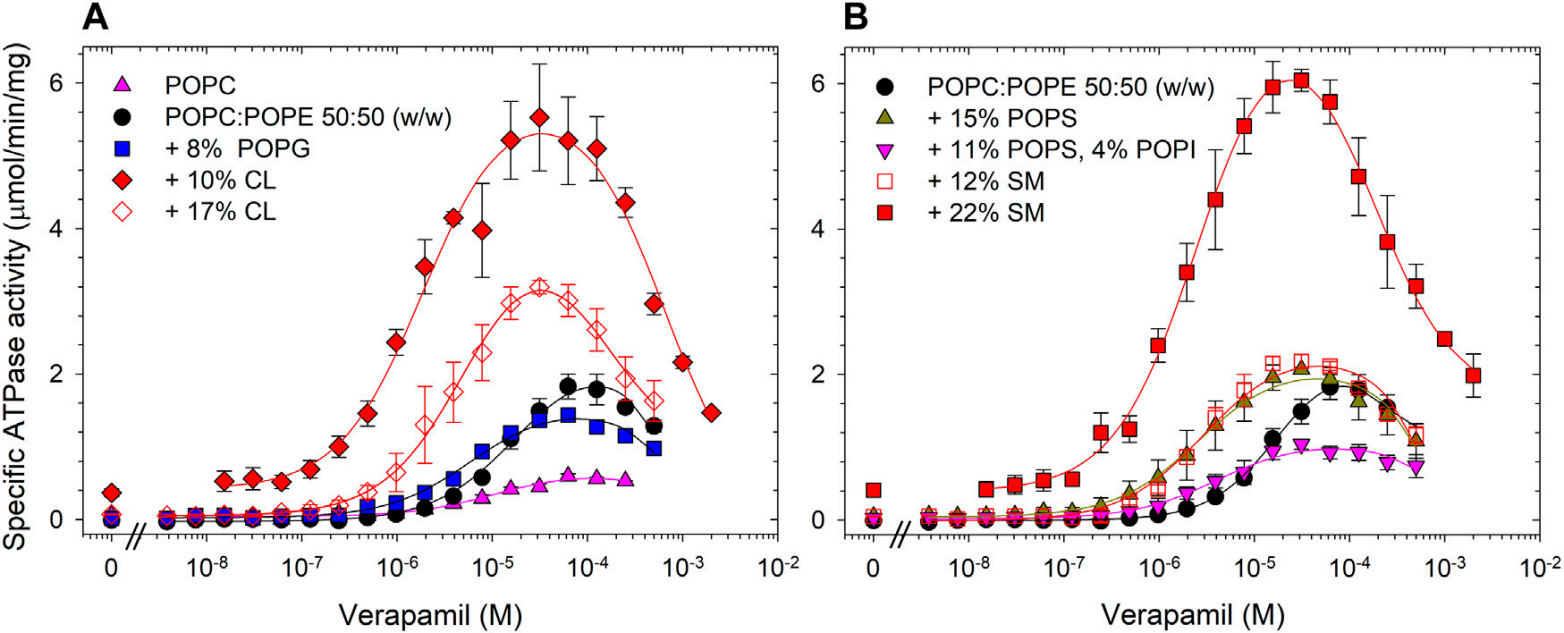
Effects of phospholipid head groups on the verapamil-stimulation of ATPase. **(A)** Lipid components of *E. coli* polar lipids, and **(B)** phospholipids found in mammalian plasma membranes. Purified Pgp was activated with 10 mM DTT and 1% POPC:POPE (50:50, w/w) lipid supplemented with indicated phospholipids as described in Experimental Procedures. Data points represent the mean ± SEM (*n* = 3). Kinetic parameters obtained from fits (solid lines) to the data points are listed in Table 2.

The first specific aim of this study was to create an artificial lipid mixture that can better mimic the mammalian plasma membrane composition but still promote Pgp ATPase activity in the same way that *E. coli* lipids can. While there is a large variety of phospholipids, the major species of fatty acids in phospholipids of the mammalian cell membranes are 16:0-18:1 (Kalvodova et al., 2009). As to the phospholipid head group, two major phospholipids found in plasma membranes are PC and PE (Table 1). Thus, the synthetic phospholipids POPC and POPE were chosen as basis to analyze the effect of individual phospholipids on Pgp ATPase activity. Purified Pgp treated with POPC alone yielded low activity. With the addition of POPE to POPC at a weight ratio of 1 to 1, half the maximum activity was reached compared to *E. coli* lipids (Figure 2; Table 2). Supplementing POPC/POPE with 10% cardiolipin increased V_max_ more than 2.5-fold but higher concentrations (17%) reversed this effect. The addition of 22% sphingomyelin also increased V_max_ significantly.

**TABLE 2:** POPC:POPE (50:50, w/w) was supplemented with indicated phospholipids and the verapamil stimulation of ATPase activity was assayed (*n* = 3).

POPC:POPE supplemented with	*V*_max_ (µmol/min/mg)	EC_50_ (µM)	Hill coefficient	Basal activity (µmol/min/mg)
POPC only	1.30 ± 0.3	6.5 ± 1.3	1.1 ± 0.2	0.05
POPC:POPE	1.83 ± 0.2	11 ± 1	1.5 ± 0.1	0.10
10% Cardiolipin	5.07 ± 0.3	1.5 ± 0.3	1.2 ± 0.1	0.38
17% Cardiolipin	3.20 ± 0.1	2.8 ± 0.4	1.4 ± 0.2	0.07
12% SM	2.18 ± 0.1	2.7 ± 0.1	1.5 ± 0.1	0.04
22% SM	5.65 ± 0.2	1.8 ± 0.2	1.2 ± 0.1	0.45
15% POPS	1.80 ± 0.1	2.4 ± 0.3	1.3 ± 0.2	0.05
11% POPS, 4% POPI	0.82 ± 0.3	2.7 ± 0.4	1.3 ± 0.2	<0.01
8% POPG	1.25 ± 0.1	4.8 ± 0.5	1.3 ± 0.2	0.05
2% DPPA	3.64 ± 0.2	0.81 ± 0.1	1.3 ± 0.2	0.83
6% DPPA	3.00 ± 0.4	0.73 ± 0.04	1.3 ± 0.1	0.16
*E. coli* polar lipids	3.65 ± 0.07	3.9 ± 0.3	1.2 ± 0.1	0.14

Importantly, addition of cardiolipin as well as SM and the acidic lipid POPS to POPC/POPE all significantly decreased the EC_50_ to around 3 μM, a value similar to *E. coli* lipids. The largest decrease in EC_50_ was observed when POPC/POPE was supplemented with the negatively charged free acid DPPA. POPS alone had little effect on V_max_, but when combined with the polar head group lipid POPI, the activity was reduced. Pgp activity was also reduced by the acidic lipid POPG. Taken together, the data demonstrate that the lipid head group plays a key role in the interaction of Pgp with verapamil, affecting V_max_ and its apparent affinity for drugs, as manifested in the effects on EC_50_ for verapamil.

### Cholesterol and CHS strongly affect Pgp ATPase activity

Cholesterol is a key component of mammalian lipid bilayers, accounting for up to 50% of the plasma membrane lipid mass (see Table 1). Biochemical studies using cholesterol, on the other hand, can be cumbersome due to its very hydrophobic nature. The hemisuccinate ester of cholesterol, CHS, is more water-soluble and therefore more suitable for many biochemical applications. CHS has been shown to be able to mimic cholesterol function (Kulig et al., 2014). Here, we examined the effects of both cholesterol and CHS on the ATPase function of Pgp.

**FIGURE 3:**
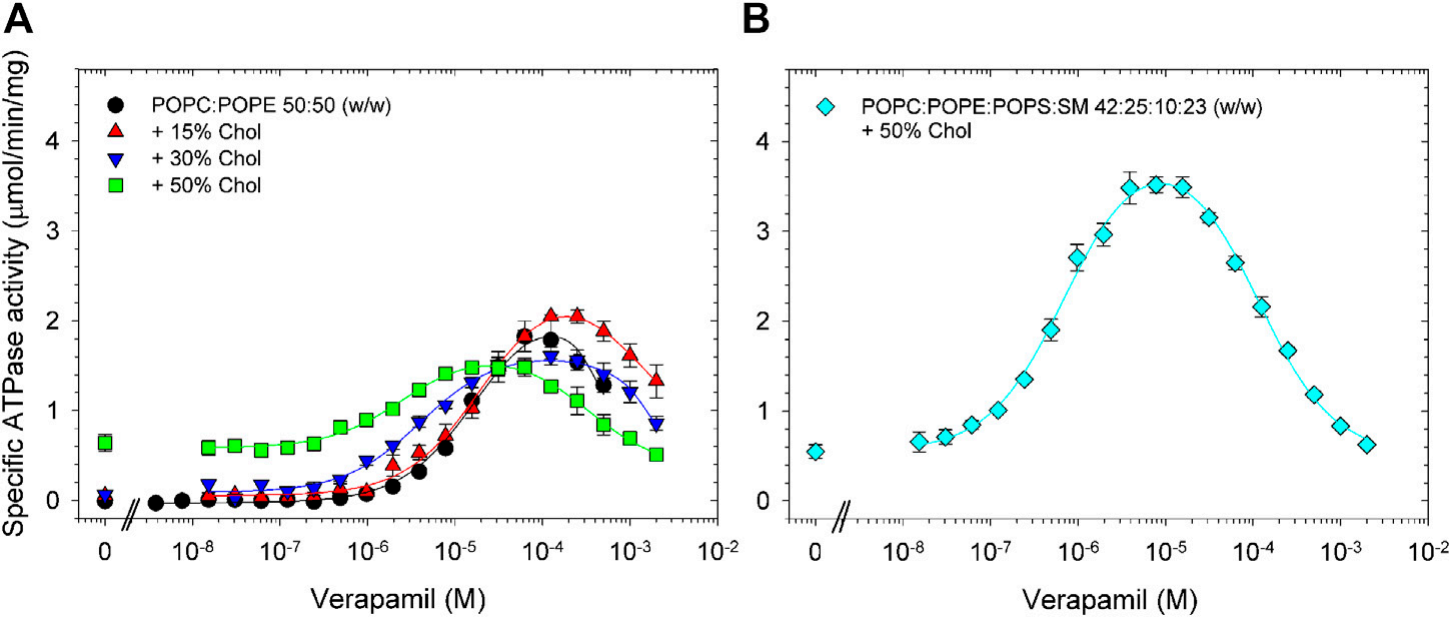
Effects of cholesterol on the verapamil-stimulation of ATPase. Purified Pgp was activated with 10 mM DTT and **(A)** POPC:POPE (50:50, w/w) lipid supplemented with 15% (w/w), 30% or 50% cholesterol. **(B)** A POPC:POPE:POPS:SM mixture (42:25:10:23, w/w) plus 50% cholesterol was used. ATPase activity was assayed as described in Experimental Procedures. Data points represent the mean ± SEM (*n* = 3).

We found that supplementing POPC/POPE lipid with 15% cholesterol had little effect on the verapamil-stimulation of the Pgp ATPase activity and on the EC_50_. Addition of 30% or 50% cholesterol to POPC/POPE decreased V_max_ moderately and gradually reduced EC_50_ (Figure 3A; Table 3). A plasma membrane mimic composition was created using POPC:POPE:POPS:SM at a ratio of 42:25:10:23 (w/w) with 50% cholesterol. The mixture was able to produce an activity similar to *E. coli* lipids while decreasing the EC_50_ by more than 20-fold (Figure 3B; Table 3). Interestingly, in both cases where 50% cholesterol was used the basal activity (in the absence of verapamil) increased.

**TABLE 3:** POPC:POPE (50:50, w/w) was supplemented with indicated concentration of cholesterol and the verapamil stimulation of ATPase activity was assayed (*n* = 3).

POPC:POPE supplemented with	*V*_max_ (µmol/min/mg)	EC_50_ (µM)	Hill coefficient	Basal activity (µmol/min/mg)	Verapamil concentration for maximum activity (µM)
None	1.9 ± 0.1	13 ± 1	1.5 ± 0.1	<0.1	60
15% Chol	2.0 ± 0.1	14 ± 1	1.1 ± 0.1	0.1	125
30% Chol	1.5 ± 0.1	3.8 ± 0.1	1.1 ± 0.1	0.1	125
50% Chol	1.2 ± 0.2	1.9 ± 0.2	1.2 ± 0.2	0.6	30
POPC:POPE:POPS:SM+ 50% Chol	3.5 ± 0.1	0.52 ± 0.06	1.2 ± 0.1	0.7	10

**FIGURE 4:**
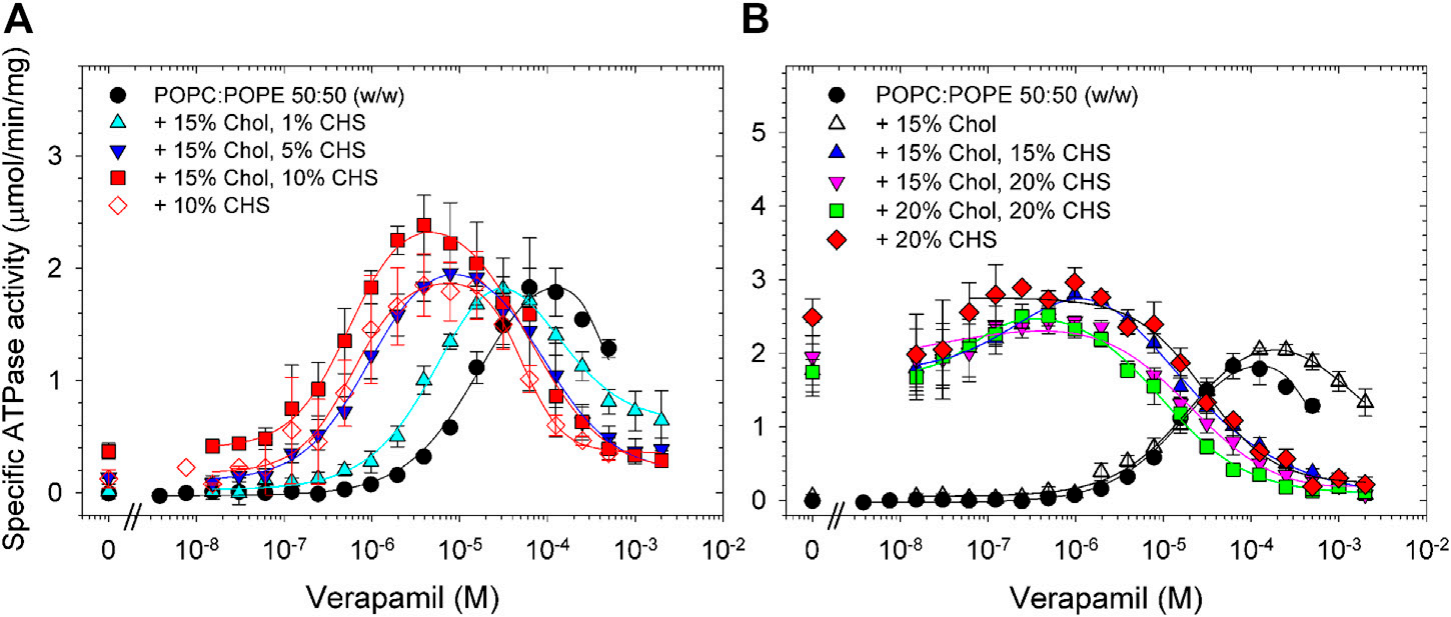
Effects on cholesterol and CHS on the verapamil-stimulation of ATPase. Purified Pgp was activated with 10 mM DTT and POPC:POPE (50:50, w/w) supplemented with the indicated concentrations of cholesterol and CHS. **(A)** 1%–10% CHS; **(B)** 15% or 20% CHS. ATPase activity was assayed as described in Experimental Procedures. Data points represent the mean ± SEM (*n* = 3).

If POPC/POPE with 15% cholesterol was additionally supplemented with as little as 1% CHS, a left-shift in the verapamil dose-response curve was observed. The decrease in EC_50_ was even more pronounced upon increasing the CHS concentration to 5% or 10%, reflecting a 20-fold increase in apparent affinity for verapamil (Figure 4A; Table 4). Interestingly, the effect of 10% CHS could even be observed in absence of cholesterol.

**TABLE 4:** POPC:POPE (50:50, w/w) was supplemented with the indicated concentrations of cholesterol and CHS and the verapamil stimulation of ATPase activity was assayed (*n* = 3).

POPC:POPE supplemented with	*V*_max_ (µmol/min/mg)	EC_50_ (µM)	Hill coefficient	Basal activity (µmol/min/mg)	Verapamil concentration for maximum activity (µM)
None	1.9 ± 0.1	13 ± 1	1.5 ± 0.1	<0.1	60
10% CHS	1.9 ± 0.2	0.49 ± 0.09	1.6 ± 0.2	0.2	4
20% CHS	3.0 ± 0.2	ND^a^	ND^a^	2.5	0.1
15% Chol, 1% CHS	1.8 ± 0.1	3.7 ± 0.3	1.5 ± 0.2	0.1	30
15% Chol, 5% CHS	1.9 ± 0.1	0.76 ± 0.06	1.3 ± 0.1	0.1	8
15% Chol, 10% CHS	2.4 ± 0.1	0.63 ± 0.09	1.5 ± 0.2	0.3	4
15% Chol, 15% CHS	2.8 ± 0.1	ND^a^	ND^a^	1.8	1
15% Chol, 20% CHS	2.4 ± 0.1	ND^a^	ND^a^	2.0	0.1
20% Chol, 20% CHS	2.5 ± 0.1	ND^a^	ND^a^	1.7	0.5

^a^ For samples containing 15% CHS, or more the basal activity (in the absence of verapamil) was high and an EC_50_ or Hill coefficient could not be determined (ND).

Increasing the CHS concentrations to 15% or 20%, in the absence or presence of up to 20% cholesterol, dramatically increased the basal activity to values much higher than those seen with cholesterol, no longer allowing the determination of EC_50_ values with certainty (Figure 4B; Table 4). Maximum activity was observed between 0.1 and 1 µM verapamil, concentrations roughly 100-fold lower than in absence of CHS. Taken together, these results show that CHS, either alone or in combination with cholesterol, substantially increases the apparent affinity of Pgp for verapamil. Interestingly, CHS also improves the thermostability of Pgp significantly (Supplementary Figure S1), as has been demonstrated for cholesterol (Rothnie et al., 2001; Bucher et al., 2007; Sharom, 2008).

### Increasing the bilayer POPC content

Since POPE does not naturally form bilayers, we explored increasing the concentration of POPC to better promote membrane formation and repeated the experiments by supplementing with either cholesterol alone or cholesterol plus CHS (see Supplementary Figure S2, Supplementary Table S1). Trends with POPC:POPE at a ratio of 80:20 (w/w) were similar as with POPC:POPE at a ratio of 50:50 (w/w). However, the higher POPC:POPE ratio of 80:20 (w/w) resulted in lower V_max_ and higher EC_50_ values as compared to the 50:50 ratio (w/w); the latter was chosen for all subsequent experiments.

### Reconstitution of Pgp-discs

The second aim of this study was to reconstitute Pgp into lipid nanodiscs with high recovery. Since the invention of nanodisc technology, protocols for reconstitution of Pgp have been reported with variations in the nanodisc size (MSP1D1 or MSP1E3D1), the lipid source, and the ratio of the components during assembly (Ritchie et al., 2009; Zoghbi et al., 2017; Alam et al., 2019; Clouser et al., 2021). Typically, to facilitate the insertion of a protein into lipid bilayers, a lipid mixture is dissolved in detergent, mixed with the protein of interest and MSP, and then the detergent is removed using Bio-Beads to initiate reconstitution. After detergent removal, Pgp-discs can be isolated from “empty” nanodiscs devoid of Pgp by affinity chromatography taking advantage of engineered purification tags on Pgp. To track recovery of Pgp-discs we recorded the verapamil-stimulated ATPase activity and quantitate the Pgp protein bands on SDS-gels before and after purification on Strep-tactin resin.

### Dissolving the lipid

First, we tested different DDM:lipid ratios from 1:1 to 8:1 (w/w) to dissolve the POPC/POPE 50:50 (w/w) mix with 15% cholesterol and 5% CHS. After detergent removal using Bio-Beads, we tracked Pgp recovery using its ATPase function. A range of DDM/lipid ratios from 1:1 to 6:1 (w/w) were effective in completely dissolving the lipid and resulted in good recovery of ATPase activity with a maximum observed at the ratio of 4:1 DDM:lipid (w/w). Increasing the DDM/lipid ratio to 7:1 or more reduced the recovered ATPase activity (Supplementary Figure S3).

#### Amount of bio-beads

Second, we tested the amount of Bio-Beads needed for maximum recovery. While Bio-Beads are effective in removing detergent, excess amounts of Bio-Beads may rapidly deplete the detergent causing the protein to precipitate before it is inserted into the lipid disc (Rothnie et al., 2001). On the other hand, not using sufficient amounts of Bio-Beads can leave traces of detergent in the solution which may bind to the drug binding sites and alter the ATPase activity (Shukla et al., 2017; Zoghbi et al., 2017). Therefore, finding the optimal amount of Bio-Beads is crucial to protein recovery and Pgp function. We tested four different ratios of Bio-Beads of 1, 2, 3, and 4 mg per 1 mg of purified Pgp. 2 mg of Bio-Beads per 1 mg Pgp produced high ATPase activity while increasing the ratio of Bio-Beads to Pgp diminished the yield of Pgp-discs. Of note, as the concentration of Bio-Beads was increased, larger protein pellets, indicative of protein precipitation, were observed concomitant with a decrease in recovered ATPase activity (Supplementary Figure S4A). Recovery of ATPase activity at 2 mg Bio-Beads per mg Pgp was around 24% ± 6% and agreed well with the recovery of the Pgp protein band of Pgp-discs resolved on Coomassie Blue-stained SDS-gels which ranged from 20% to 25% (Supplementary Figure S4B).

#### Ratio of MSP to phospholipids

To assemble Pgp-discs, two membrane scaffold proteins (MSPs) are needed to enclose one Pgp molecule in a lipid nanodisc. Using excess MSP will increase the number of “empty” nanodiscs devoid of Pgp, while excess lipids can create Pgp proteoliposomes instead of or in addition to nanodiscs. Therefore, finding the optimal ratio of MSP to phospholipid is important. We tested a range of MSP:phospholipid ratios from 1:30 to 1:70 (mol/mol) using POPC/POPE supplemented with 15% cholesterol and 10% CHS. After Bio-Beads treatment and purification on Strep-tactin resin, the highest ATPase activity was recovered at a ratio of 1:40-1:50 of MSP to lipid (mol/mol) (Supplementary Figure S5). At an MSP:lipid ratio of 1:60 or less, recovery declined.

#### Reconstitution of Pgp in proteoliposomes and nanodiscs

Using the optimized conditions described above, we reconstituted Pgp-discs in POPC/POPE supplemented with 15% cholesterol and 0%, 10%, or 20% CHS to observe the effect of CHS on reconstitution. Purified protein was mixed with a detergent-lipid mixture and then the detergent was removed using Bio-Beads to initiate reconstitution. One sample received an addition of MSP to generate nanodiscs while the other sample without MSP will form proteoliposomes. Pgp-discs were isolated by affinity chromatography. Recovery of Pgp-discs was tracked by measuring verapamil-stimulated ATPase activity after purification on Strep-tactin resin.

**FIGURE 5:**
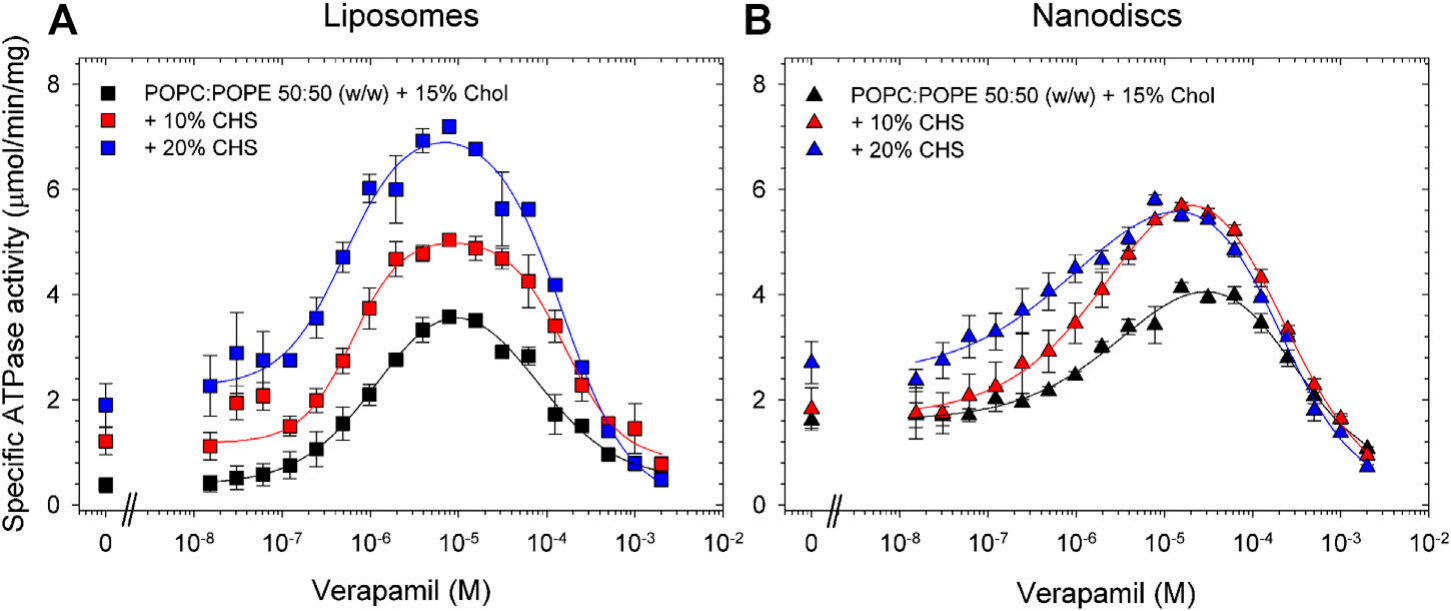
Effects of CHS on the verapamil-stimulation of ATPase in proteoliposomes and nanodiscs. Purified Pgp was reconstituted into **(A)** liposomes and **(B)** lipid nanodiscs containing POPC/POPE supplemented with 15% cholesterol and 0%, 10%, and 20% CHS. ATPase activity was measured as described in Experimental Procedures. Data points represent the mean ± SEM (*n* = 3). In liposomes, more than 86% of Pgp’s NBDs were oriented inside-out and accessible to ATP hydrolysis as inferred from accessible to cleavage of the C-terminal Strep-tag, see Fig. S7.

Compared to the results obtained with lipid-activated Pgp (Figure 4; Table 4), the reconstituted protein (Figure 5; Tables 5, 6) demonstrated overall superior function. In absence of CHS, reconstituted Pgp showed an increased ATPase activity by a factor of about 2 and a roughly 10-fold decrease in EC_50_ for verapamil activation, both in proteoliposomes and in nanodiscs. Addition of 10% or 20% CHS resulted in an increase of ATPase activity and a gradual decrease of EC_50_ in all cases, although the effect on EC_50_ was smaller for the reconstituted samples due to their already reduced EC_50_ in absence of CHS. Recovery was similar without or with CHS (Supplementary Figure S6). Compared to lipid-activated Pgp, the reconstituted samples had a higher basal activity, especially in nanodiscs.

**TABLE 5:** Pgp was reconstituted into liposomes prepared from POPC/POPE with the indicated concentrations of CHS and the verapamil stimulation of ATPase activity was assayed (*n* = 3).

POPC:POPE liposomes supplemented with	*V*_max_ (µmol/min/mg)	EC_50_ (µM)	Hill coefficient	Basal activity (µmol/min/mg)	Verapamil concentration for maximum activity (µM)
15% Chol	3.6 ± 0.1	0.89 ± 0.07	1.1 ± 0.1	0.4	8
15% Chol, 10% CHS	5.0 ± 0.1	0.69 ± 0.12	1.4 ± 0.4	1.2	8
15% Chol, 20% CHS	7.2 ± 0.1	0.48 ± 0.09	1.3 ± 0.3	1.9	8

**TABLE 6:** Pgp was reconstituted into lipid nanodiscs prepared from POPC/POPE with the indicated concentrations of CHS and the verapamil stimulation of ATPase activity was assayed (*n* = 3).

POPC:POPE nanodiscs supplemented with	*V*_max_ (µmol/min/mg)	EC_50_ (µM)	Hill coefficient	Basal activity (µmol/min/mg)	Verapamil concentration for maximum activity (µM)
15% Chol	4.1 ± 0.1	1.83 ± 0.37	1.0 ± 0.2	1.6	16
15% Chol, 10% CHS	5.7 ± 0.1	1.14 ± 0.19	1.0 ± 0.2	1.8	16
15% Chol, 20% CHS	5.8 ± 0.1	0.68 ± 0.31	0.7 ± 0.2	2.7	8

**FIGURE 6:**
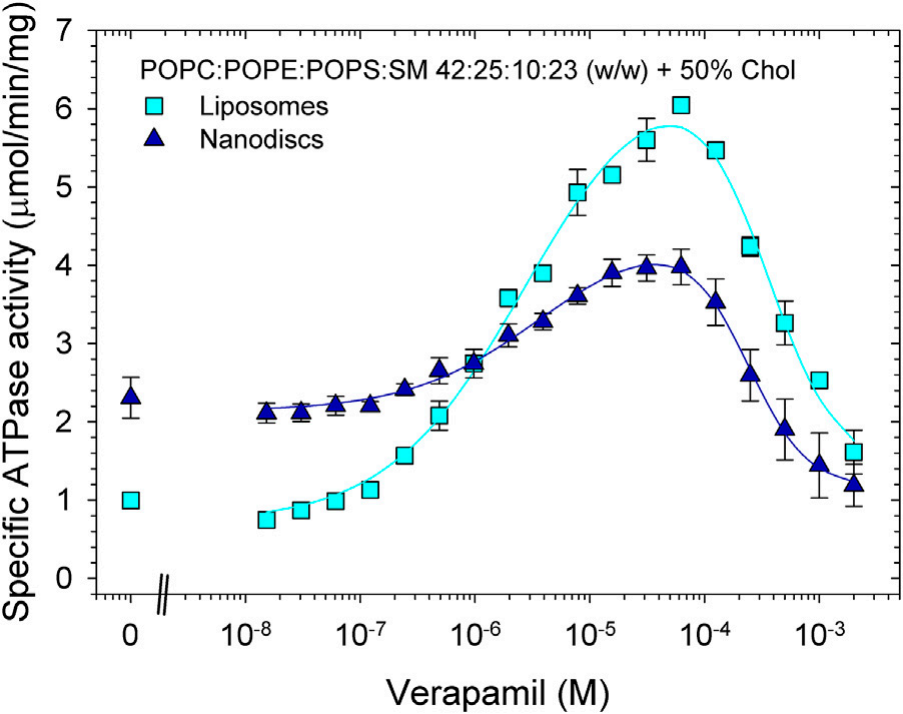
Effects of reconstitution on the verapamil-stimulation of ATPase in lipids mimicking plasma membranes. Purified Pgp was reconstituted into liposomes and lipid nanodiscs. ATPase activity was measured as described in Experimental Procedures. Data points represent the mean ± SEM (*n* = 3). In liposomes, more than 84% of Pgp’s NBDs were accessible, see Fig. S7.

Finally, we reconstituted Pgp in proteoliposomes and nanodiscs containing the phospholipid composition mimicking plasma membranes (POPC:POPE:POPS:SM at a ratio of 42:25:10:23 (w/w) with 50% cholesterol). In contrast to the samples reconstituted in POPC/POPE/cholesterol (Figure 5; Tables 5, 6), reconstitution of Pgp in plasma membrane mimic lipids, either as proteoliposomes or as nanodiscs, did not improve functionality compared to the lipid-activated protein. While the V_max_ of verapamil-stimulated ATPase activity was slightly to moderately increased, the apparent affinity for verapamil decreased by a factor of 4 (Figure 6; Table 7). Again, Pgp in nanodiscs showed a high basal activity.

**TABLE 7:** Pgp was reconstituted intp proteoliposomes and lipid nanodiscs prepared from plasma membrane mimicking lipids, and the verapamil stimulation of ATPase activity was assayed (*n* = 3).

Sample preparation	*V*_max_ (µmol/min/mg)	EC_50_ (µM)	Hill coefficient	Basal activity (µmol/min/mg)	Verapamil concentration for maximum activity (µM)
Lipid-activated	3.5 ± 0.1	0.52 ± 0.06	1.2 ± 0.1	0.7	10
Proteoliposomes	6.0 ± 0.1	2.2 ± 0.3	1.1 ± 0.1	1.0	60
Nanodiscs	4.0 ± 0.2	2.1 ± 0.4	0.7 ± 0.1	2.3	30

## Discussion

In this study, we created an artificial lipid mixture that comes closer to the mammalian plasma membrane composition but still promotes Pgp ATPase activity in the same way that *E. coli* lipids can. Our study will facilitate future investigations of the mechanism of Pgp such as using tryptophan fluorescence, in order to overcome the high background signal from natural lipid extracts (such as *E. coli* lipids). Two major components of mammalian plasma membranes are PC and PE, while *E. coli* polar lipids contain a large amount of PE, but no PC. Using a mixture of synthetic POPC and POPE in a ratio of 50:50, Pgp fell short of reaching the verapamil-stimulated ATPase activity and apparent verapamil affinity observed with *E. coli* polar lipids. However, with supplements such as cardiolipin, SM, or DPPA, the POPC/POPE mixture was able to restore Pgp functionality to the same level observed with *E. coli* polar lipids or even higher. It should be emphasized that the activities and affinities reached here are similar to those found in the natural environment of Pgp, the plasma membrane (al-Shawi and Senior, 1993; Shukla et al., 2017).

The main effect of these lipid supplements was to reduce the EC_50_ for verapamil, while verapamil-stimulated ATPase activity (V_max_) was only moderately affected, if at all. Of these three components, only SM is found in mammalian plasma membranes in significant amounts. It is not clear how these components support Pgp functionality or if they do it by the same mechanism. Cardiolipin and DPPA are negatively charged, but SM is not; furthermore, the negatively charged phospholipids POPS and POPG did not improve Pgp performance. Both DPPA and the SM preparation used in this study contain exclusively or nearly exclusively saturated fatty acids, which tend to reduce the membrane fluidity. On the other hand, the cardiolipin preparation used here carries mostly doubly-unsaturated fatty acids, which are expected to increase membrane fluidity.

Cholesterol is a major component of mammalian plasma membranes. However, adding cholesterol to the POPC/POPE mixture, even in amounts as high as 50%, did not improve Pgp functionality to a pronounced extent. While the apparent ligand binding affinity was somewhat increased, the ATPase activity dropped. In contrast, a mammalian plasma membrane mimic, containing POPC:POPE:POPS:SM at a ratio of 42:25:10:23 (w/w) with 50% cholesterol gave a verapamil-stimulated ATPase activity similar to *E. coli* lipids while increasing the apparent affinity for verapamil significantly. Taken the results described above into account, this increase in Pgp functionality is most likely due to the presence of SM, not POPS.

Another compound we identified as supportive of Pgp performance was CHS. Adding 10% CHS to a POPC/POPE mix supplemented with 15% cholesterol improved the V_max_ of verapamil-stimulated ATPase activity only slightly but increased the apparent affinity for verapamil by a factor of 20. High concentrations of CHS resulted in a significantly increased basal activity which could be due to binding of CHS to the drug binding site as transport substrate or as activator, maybe acting cooperatively with verapamil to increase the verapamil affinity. Alternatively, cholesterol has been shown by cryo-EM to bind to grooves on the surface of the TMDs of Pgp (Alam et al., 2019). CHS might do the same and stabilize Pgp in a conformation that is hydrolysis-active and has a high affinity for verapamil. The notion of direct interactions of CHS with Pgp, whether in the drug binding site or on the surface of the transmembrane region, is supported by the finding that CHS increases the thermostability of DDM-solubilized Pgp.

Following the systematic investigation of lipid effect on Pgp activity, we optimized conditions for reconstitution of Pgp in nanodiscs, especially with regard to recovery, and to see if the artificial lipid mixtures identified above also worked well in nanodiscs. The optimal DDM:lipid ratio for solubilizing the lipids ratio was determined to be 4:1 and the best ratio of Bio-Beads to Pgp was found to be 2 mg Bio-Beads per 1 mg Pgp. The MSP:lipid ratio was optimal at 1:40–1:50. Using these conditions, we reconstituted Pgp into proteoliposomes and nanodiscs using POPC/POPE with 15% cholesterol and 0%, 10% or 20% CHS. Especially when prepared in presence of CHS, the resulting proteoliposomes and nanodiscs both strongly supported Pgp functionality. Both verapamil-stimulated ATPase activity and apparent verapamil affinity were actually superior to results observed in preparations where detergent-solubilized Pgp was just mixed with lipids, without undergoing the reconstitution procedure.

When using the plasma membrane mimic lipid mixture (POPC:POPE:POPS:SM at a ratio of 42:25:10:23 (w/w) with 50% cholesterol for the reconstitution, we obtained again proteoliposomes and nanodiscs with active Pgp. However, the verapamil-stimulated ATPase activity in nanodiscs was only about half of the activity observed in proteoliposomes. While the lipid composition of the proteoliposomes likely reflects the composition of the lipid mix user for the reconstruction, this might not be the case for the nanodiscs. It could be shown that nanodiscs prepared from binary mixtures of POPC and POPG or POPS in different ratios have a lipid composition reflecting the original mixture (Hoi et al., 2016). However, these phospholipids are very similar, just differing in the head group. Experimental data on nanodiscs prepared from more mixtures of structurally more diverse lipids are lacking (Li et al., 2019). Another possible problem is a heterogeneous distribution of lipids in nanodiscs due to the presence of MSP. A molecular dynamics study found an even distribution of cholesterol in the nanodiscs, while the negatively charged CHS preferred to bind to positively charged amino acid residues in MSP (Augustyn et al., 2019). Even if some CHS binds to MSP, the results of the present study show that there must be still CHS molecules available to bind to Pgp and improve its functionality.

In conclusion, we could identify improved lipid mixtures that are able to sustain high levels of activity and verapamil binding affinities very similar to Pgp in plasma membranes. This will pave the way for future functional and biophysical studies of Pgp in a lipid environment that supports enzyme activity and mirrors the drug binding properties in its native membrane environment.

## Data Availability

The original contributions presented in the study are included in the article/Supplementary Material, further inquiries can be directed to the corresponding author.

## Nomenclature

### Abbreviations

Pgp: P-glycoprotein
ABC: ATP Binding Cassette
TMD: transmembrane domain
NBD: nucleotide binding domain
WL-MSP: tryptophan-free membrane scaffold protein
DDM: n-dodecyl-β-D-maltopyranoside
CHS: cholesteryl hemisuccinate
POPC: 1-palmitoyl-2-oleoyl-sn-glycero-3-phosphocholine or 16:0-18:1 PC
POPE: 1-palmitoyl-2-oleoyl-sn-glycero-3-phosphatidylglycerol
POPS: 1-palmitoyl-2-oleoyl-sn-glycero-3-phospho-L-serine
POPI: 1-palmitoyl-2-oleoyl-sn-glycero-3-phosphatidylinositol
POPG: 1-palmitoyl-2-oleoyl-sn-glycero-3-phosphatidylethanolamine
SM: sphingomyelin
DPPA: 1,2-dipalmitoyl-sn-glycero-3-phosphate or 16:0 PA
CL: cardiolipin
TEV: tobacco etch virus protease
