# Supplementary material for: Lipid environment determines the drug-stimulated ATPase activity of P-glycoprotein
Source: Front Mol Biosci. 2023 Feb 23;10:1141081. doi: 10.3389/fmolb.2023.1141081 (PMC9995911; doi:10.3389/fmolb.2023.1141081)
Supplement: Supplementary file 1 [file DataSheet1.pdf]

Supplementary information

Lipid environment determines the drug-stimulated ATPase activity of P-glycoprotein

Nghi N.B. Tran[1,2], Anthony T.A. Bui[1,2], Valeria Jaramillo-Martinez[1,2], Joachim Weber[2,3], Qinghai Zhang[4] and Ina L. Urbatsch[1,2]*

From the [1]Department of Cell Biology and Biochemistry, the [2]Center for Membrane Protein Research, Texas Tech University Health Sciences Center, Lubbock, Texas, USA, the [3]Department of Chemistry and Biochemistry, Texas Tech University, Lubbock, Texas, USA, and the [4]The Scripps Research Institute, La Jolla, California, USA

Short title: *Lipids stimulate the P-glycoprotein ATPase*

***)** To whom correspondence may be addressed: Ina L. Urbatsch, Department of Cell Biology and Biochemistry, Texas Tech University Health Sciences Center, Lubbock, TX, 79430, USA; Tel. +1 (806) 743-1192, E-Mail: ina.urbatsch@ttuhsc.edu;

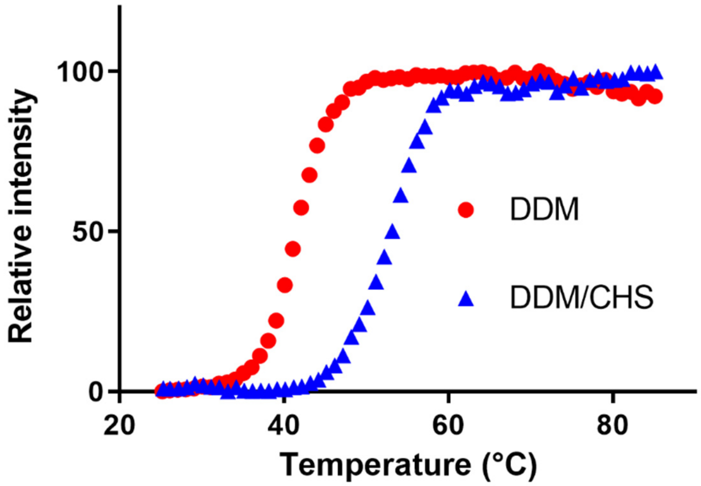

Figure S1: CHS increases the thermostability of purified Pgp in DDM detergent solution. Pgp was purified from microsomal membranes as detailed in Methods in either 0.05% DDM or 0.05% DDM containing 0.01% CHS (DDM/CHS). Samples were incubated with 2 µM of the cysteine-reactive dye 7-Diethylamino-3-(4'-maleimidylphenyl)-4-methylcoumarin (CPM), and the increase in fluorescence upon heating the samples with a temperature ramp of 1ºC/min was monitored with excitation/ emission wavelengths of 384/470 nm. Increase in fluorescence is due to protein unfolding and exposure of reactive cysteines in Pgp. The melting temperature T_m was ~40ºC for Pgp in DDM solution, similar to that reported for Pgp by other methods (Yang et al., 2017). T_m of Pgp in DDM/CHS was significantly increased to 52ºC. Representatives of three independent experiments are shown.

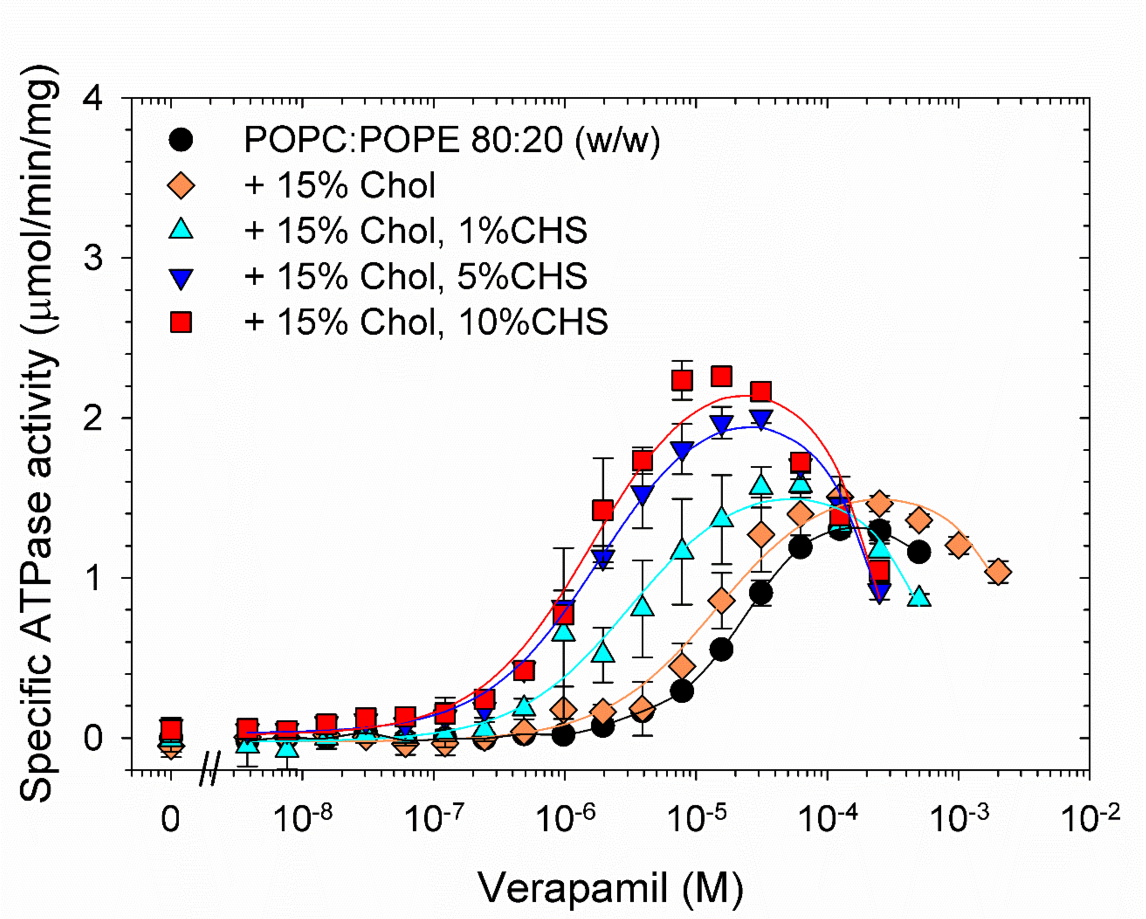

Figure S2: Effects of cholesterol and its hemisuccinate ester (CHS) on the verapamil stimulation of Pgp ATPase activity. Purified Pgp was activated with 10 mM DTT and 1% POPC:POPE (80:20, w/w) lipids supplemented with 15% cholesterol and increasing amounts of CHS as indicated. ATPase activity was assayed as in Figure 4.

Table S1: POPC:POPE (80:20, w/w) was supplemented with indicated cholesterol and CHS and the kinetics of the verapamil stimulation of ATPase activity determined from data in Figure S1 (n=3).

POPC:POPE supplemented with	V_{max} (µmol/min/mg)	EC_{50} (µM)	Basal activity (µmol/min/mg)	Verapamil concentration for maximum activity (µM)
None	1.3 ± 0.1	17 ± 1	< 0.1	125
15% Chol	1.5 ± 0.1	12 ± 1	< 0.1	125
15% Chol, 1% CHS	1.6 ± 0.1	2.7 ± 0.6	< 0.1	60
15% Chol, 5% CHS	1.7 ± 0.1	1.4 ± 0.2	0.1	60
15% Chol, 10% CHS	2.2 ± 0.1	1.6 ± 0.1	0.1	8

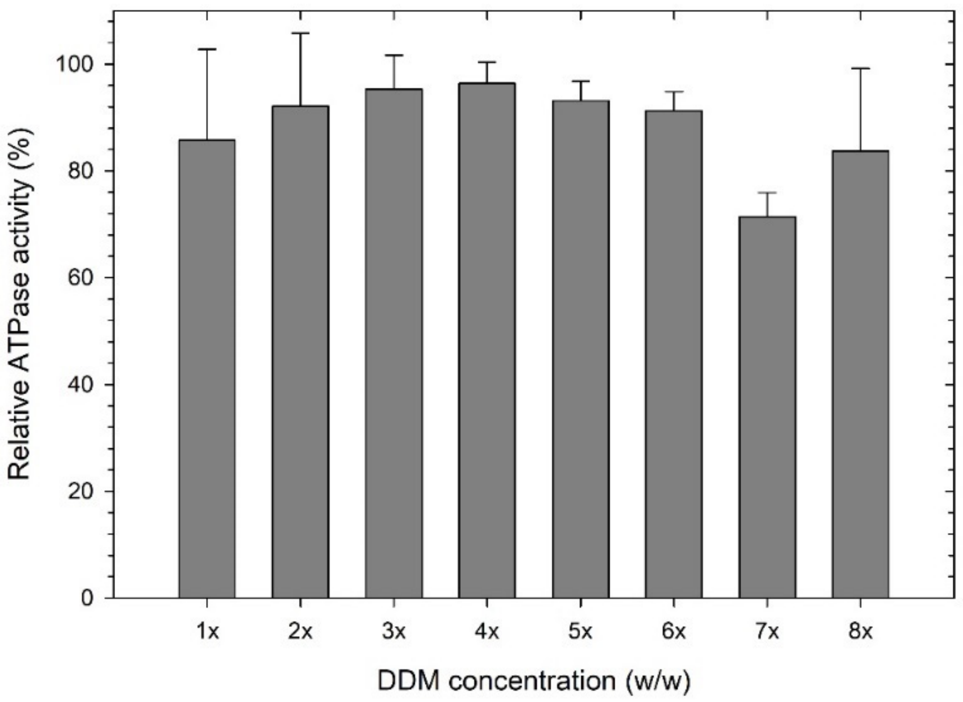

Figure S3: Effect of increasing DDM concentrations on dissolving lipids for reconstitutions. POPC/POPE 50:50 mix containing 15% Chol (w/w) was dissolved in DDM at increasing DDM:phospholipid ratios. Dissolved lipids were then mixed with purified Pgp and MSP1E3D1 at a ratio of 1:5 (mol/mol) and MSP:phospholipid of 1:50 (mol/mol) and incubated with 2 mg Bio-Beads per mg Pgp for 16 h. Samples were centrifuged at 14,000 g for 15 min at 4ºC to remove precipitated protein. These samples contain mixtures of proteoliposomes and nanodoscs, and were assayed for ATPase activity without further separation.

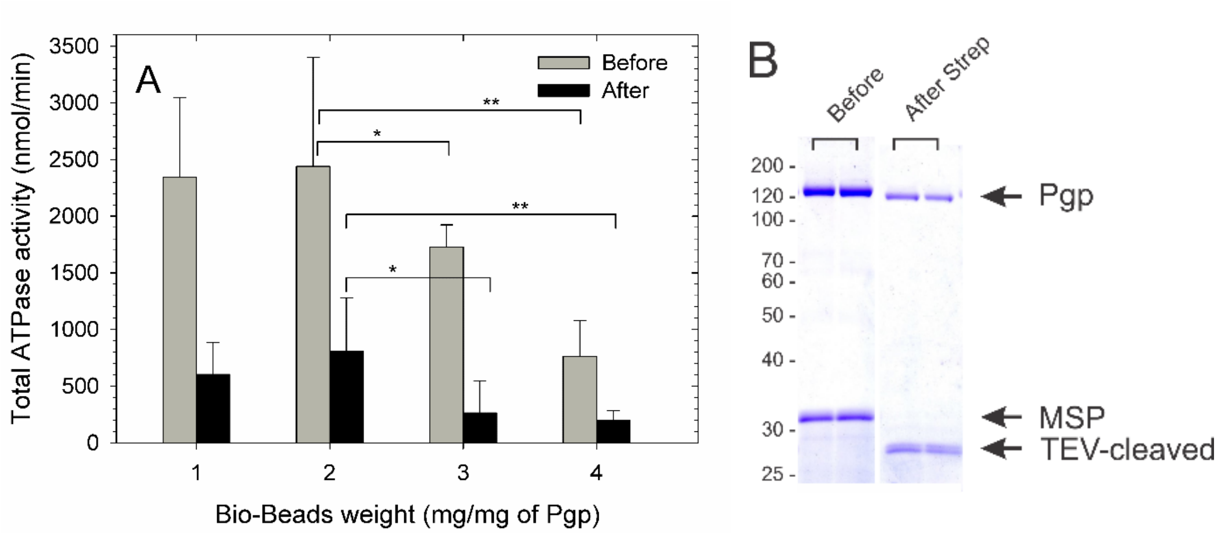

Figure S4: Recovery of total ATPase activity and Pgp protein before and after separation of Pgp-discs on Strep-tactin resin. A POPC/POPE 50:50 mix containing 15% Chol (w/w) was dissolved in DDM at a DDM:phospholipid ratio of 4:1 (w/w). 1 mg of Pgp was mixed with MSP1E3D1 and phospholipid (assuming an average molecular weight of 752 g/mol) using a molar ratio of Pgp:MSP of 1:5 (mol/mol) and MSP:phospholipid of 1:50 (mol/mol) and incubated with increasing amounts of Bio-Beads (1 to 4 mg per mg Pgp) for 16 h. After centrifugation to remove precipitated protein, the Pgp-nanodiscs were assayed for ATPase activity (labeled "Before"). Then Pgp-discs were purified by binding to Strep-tactin resin via Pgp's C-terminal Twin-Strep tag, empty nanodiscs devoid of Pgp were washed off, and Pgp-nanodiscs were eluted by cleavage of the Twin-Strep tag with Tobacco Etch Virus protease (labeled "After"). (A) Recovery of ATPase activity before and after separation on Strep-tactin was assayed in the presence of 30 μM verapamil; total activity per 1 mg Pgp starting material is given. Data points represent the mean ± SEM (n=3-6). Data was statistically analyzed by two-way ANOVA followed by Bonferroni test; a p-value of < 0.05 was considered significant (*) and $P < 0.01$ (**). (B) Protein samples (volume equivalents) from the best condition (2mg Bio-Beads per mg Pgp) were resolved on a 10% SDS-gel and stained with Coomassie-blue, and recovery of Pgp protein was quantitated by ImageJ. Pgp protein recovery closely matched the recovery of ATPase activity and was about 33 ± 19% of the input Pgp in this experiment using 2 mg/mg Bio-Beads:Pgp. At Bio-Beads amounts of 3 mg or more per mg Pgp recovery diminished, possibly because Pgp absorbed to the hydrophobic beads.

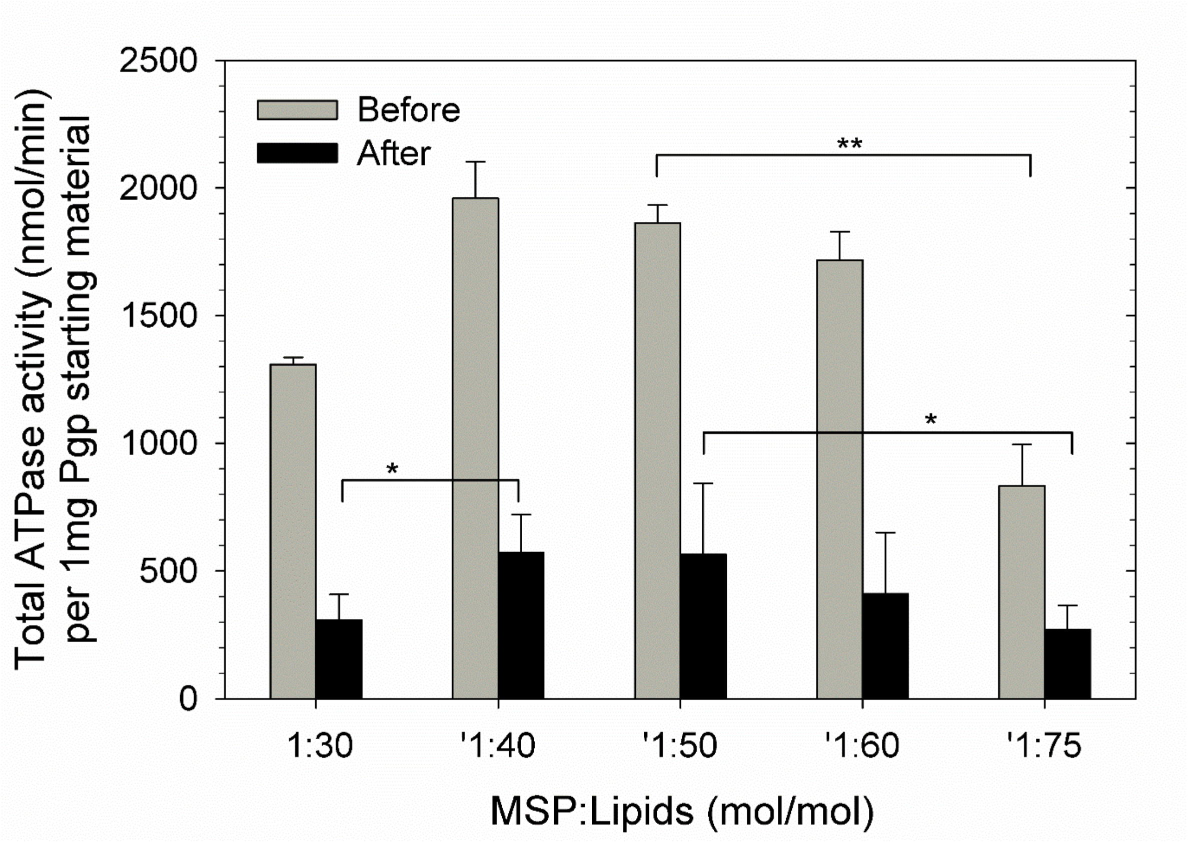

Figure S5: MSP to phospholipid ratio. Recovery of total ATPase activity and Pgp protein before and after separation of Pgp-discs on Strep-tactin resin was assayed as in figure 4, except that nanodisc formation was induced using increasing ratios of MSP:phospholipid. The best recovery of ATPase activity was seen at ratios of 1:40 to 1:50 MSP:phospholipid. Data was statistically analyzed by two-way ANOVA followed by Bonferroni test.

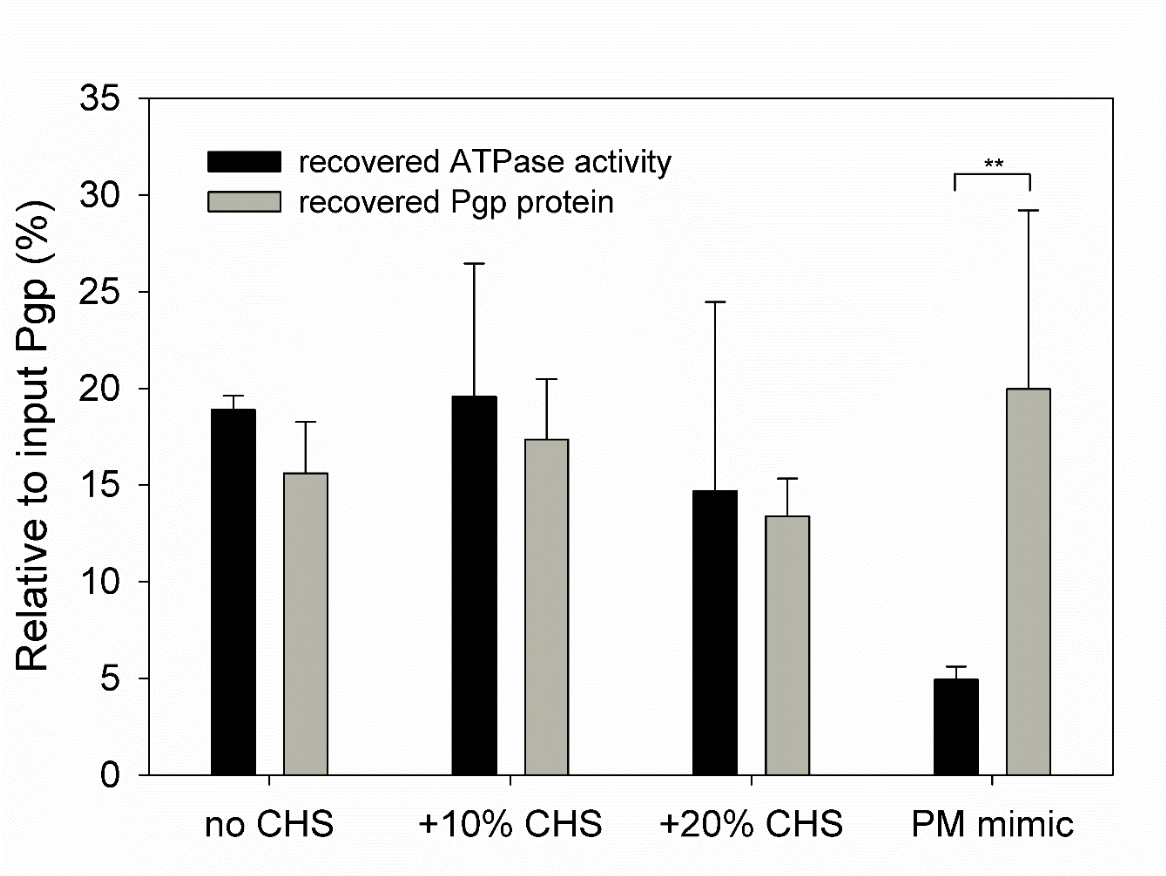

Figure S6: Recovery of Pgp in nanodiscs with either POPC/POPE containing cholesterol and indicated concentrations of 0, 10% and 20% CHS (leftmost bars), or with PM lipid mimic (right bars). Inclusion of increasing concentrations of CHS into the POPC/POPE + 15% cholesterol lipid mix used for formation of nanodiscs did not significantly affect the recovery of Pgp in Pgp-discs as assayed by ATPase activity or by quantitation of the Pgp protein bands on Coomassie-stained SDS-gels. Recovery of Pgp-discs assembled with plasma membrane (PM) lipid mimic showed low recovery of ATPase activity, albeit Pgp protein recovery appeared normal.

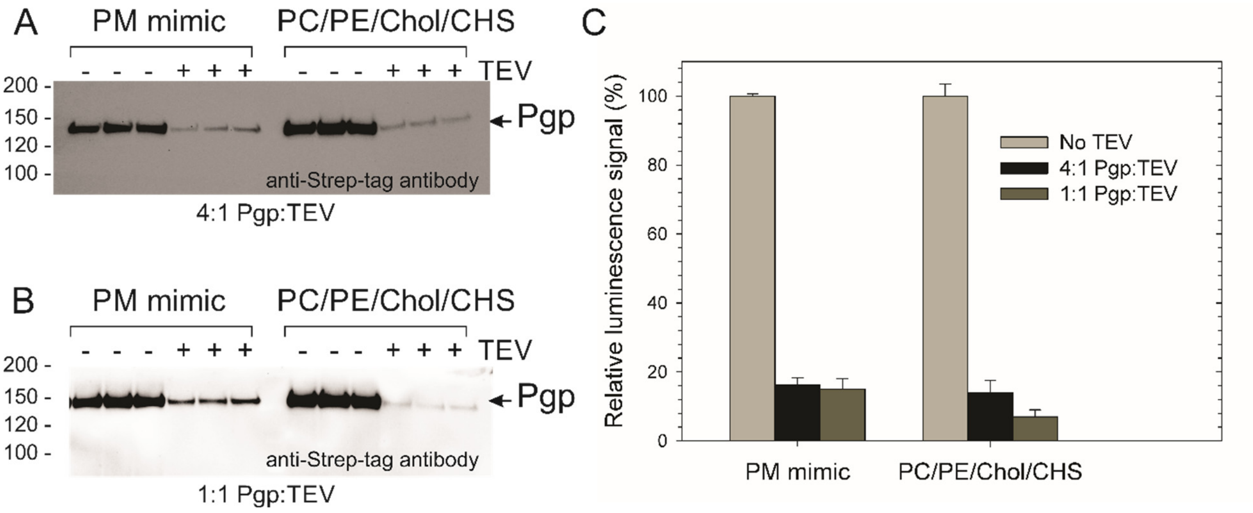

Figure S7: Accessibility of Pgp in liposomes. To investigate the orientation of Pgp in proteoliposomes we took advantage of the TEV-cleavable Strep-tag at the C-terminal end of NBD2 of Pgp to probe accessibility to the potease in sealed liposomes. Pgp-liposomes were incubated overnight at 4C without and with TEV protease at a ratio of A) 4:1 (w/w) Pgp:TEV and B) 1:1 (w/w) Pgp:TEV, then samples were resolved on SDS-gels and subjected to Western blot analysis using anti-Strep-tag antibody. Both Pgp liposomes formed with the plasma membrane (PM) mimic lipid POPC:POPE:POPS:SM (42:25:10:23, w/w) with 50% cholesterol, and liposomes formed with POPC/POPE containing 15% cholesterol and 10% CHS (PC/PE/Chol/CHS) were analysed. In PM mimic liposomes, 84 ± 2% and 85 ± 3 % of NBDs were oriented inside-out and accessible to TEV cleave. This number was higher in PC/PE/Chol/CHS liposomes with 86 ± 4% and 93 ± 2% accessible.

Supplemental references

Yang, Z., Zhou, Q., Mok, L., Singh, A., Swartz, D.J., Urbatsch, I.L., et al. (2017). Interactions and cooperativity between P-glycoprotein structural domains determined by thermal unfolding provides insights into its solution structure and function. *Biochim Biophys Acta Biomembr* 1859(1), 48-60. doi: 10.1016/j.bbamem.2016.10.009.
